# Novel method to rescue a lethal phenotype through integration of target gene onto the X-chromosome

**DOI:** 10.1038/srep37200

**Published:** 2016-11-15

**Authors:** Kazuya Sakata, Kimi Araki, Hiroyasu Nakano, Takashi Nishina, Sachiko Komazawa-Sakon, Shin Murai, Grace E. Lee, Daisuke Hashimoto, Chigure Suzuki, Yasuo Uchiyama, Kenji Notohara, Anna S. Gukovskaya, Ilya Gukovsky, Ken-ichi Yamamura, Hideo Baba, Masaki Ohmuraya

**Affiliations:** 1 Institute of Resource Development and Analysis, Kumamoto University, 2-2-1 Honjo, Chuo-ku, Kumamoto 860-0811, Japan; 2 Department of Gastroenterological Surgery, Kumamoto University, 1-1-1 Honjo, Chuo-ku, Kumamoto 860-0811, Japan; 3 Department of Biochemistry, Toho University School of Medicine, 5-21-16 Omori-Nishi, Ota-ku, Tokyo 143-8540, Japan; 4 Department of Medicine, David Geffen School of Medicine at the University of California Los Angeles, and VA Greater Los Angeles Healthcare System, Los Angeles, CA, USA; 5 Department of Cellular and Molecular Neuropathology, Juntendo University Graduate School of Medicine, 2-1-1 Hongo, Bunkyo-Ku, Tokyo 113-8421, Japan; 6 Department of Pathology, Kurashiki Central Hospital, 1-1-1 Miwa, Kurashiki, Okayama 710-8602, Japan.

## Abstract

The loss-of-function mutations of serine protease inhibitor, Kazal type 1 (*SPINK1*) gene are associated with human chronic pancreatitis, but the underlying mechanisms remain unknown. We previously reported that mice lacking *Spink3*, the murine homologue of human *SPINK1*, die perinatally due to massive pancreatic acinar cell death, precluding investigation of the effects of SPINK1 deficiency. To circumvent perinatal lethality, we have developed a novel method to integrate human *SPINK1* gene on the X chromosome using Cre-*lox*P technology and thus generated transgenic mice termed “*X-SPINK1*“. Consistent with the fact that one of the two X chromosomes is randomly inactivated, *X-SPINK1* mice exhibit mosaic pattern of *SPINK1* expression. Crossing of *X-SPINK1* mice with *Spink3*^+*/*−^ mice rescued perinatal lethality, but the resulting *Spink3*^−*/*−^*;XX*^*SPINK1*^ mice developed spontaneous pancreatitis characterized by chronic inflammation and fibrosis. The results show that mice lacking a gene essential for cell survival can be rescued by expressing this gene on the X chromosome. The *Spink3*^−/−^*;XX*^*SPINK1*^ mice, in which this method has been applied to partially restore SPINK1 function, present a novel genetic model of chronic pancreatitis.

Trypsin is a major serine protease produced in pancreatic acinar cells as inactive zymogen (trypsinogen). In physiological conditions, trypsinogen is secreted by the acinar cells and is cleaved/activated in the duodenum by *enterokinase*, resulting in generation of trypsin^1^,^2^. Human serine protease inhibitor, Kazal type 1 (*SPINK1*) and its murine homologue *Spink3* play a critical role in suppression of aberrant, intra-acinar/intrapancreatic activation of trypsinogen, which is considered a key mechanism preventing the development of pancreatitis^3^,^4^. Consistent with this concept, loss-of-function mutations of *SPINK1* gene are associated with various forms of human chronic pancreatitis; however, the mechanisms through which *SPINK1* mutations predispose to pancreatitis remain elusive^5^,^6^. We have previously reported^3^ that *Spink3*^−*/*−^ mice spontaneously develop severe pancreatic damage and die within two weeks after birth. The histopathological changes start gradually at embryonic day (E) 16.5 and are restricted to pancreatic acinar, but not ductal or islet, cells. The cytoplasm of acinar cells of *Spink3*^−*/*−^ mice is filled with numerous autophagic vacuoles^3^, suggesting that *Spink3* deletion interferes with autophagy, a key cellular, lysosome-driven process that degrades and recycles damaged or unneeded organelles, long-lived proteins, and lipids^7^. The aberrant autophagy could trigger acinar cell death in *Spink3*^−*/*−^ mice (it is, however, worth noting that these cells do not display chromatin condensation, a hallmark of apoptosis)^3^. The early death of *Spink3*^−*/*−^ mice precludes investigation into the mechanisms of long-term effects of SPINK1 deficiency.

X-chromosome inactivation is a process by which one of the two X chromosomes in female mammals is randomly inactivated by packaging into transcriptionally inactive heterochromatin^8^. Once the X chromosome is inactivated, it will remain inactive throughout the lifetime of the cell. During screening of a gene trap library, we found that one ES cell line (B210) possessed a trap vector on the X chromosome. By utilizing X-chromosome inactivation and the B210 ES cells, we here present a novel method to efficiently integrate a target gene on the X chromosome, resulting in a mosaic pattern of the target protein expression. This strategy enabled us to rescue perinatal lethality of *Spink3*^−*/*−^ mice. The resulting *Spink3*^−*/*−^*;XX*^*SPINK1*^ mice developed spontaneous pancreatitis, thus representing a novel genetic model for this disease.

## Results

### Integration of *SPINK1* gene at a locus on X chromosome by using Cre-*lox*P technology results in a mosaic pattern of its expression

Gene-trap mutagenesis is a technique that randomly generates loss-of-function mutations in many genes^9^. During gene-trap mutagenesis screening, we found that in one ES cell line (designated B210 ES cells) the integrated trap vector inactivated *Diaphanous homolog 2 (Diap2*) gene on the X chromosome^10^ (Fig. S1a). *Diap2,* also known as murine *Dia3,* is one of three members of the *Diap* family and considered to play a role in *de novo* actin filament formation^11^. Subsequent plasmid rescue and PCR analysis revealed that the trap vector was integrated into intron 23 of the *Diap2* gene at 50 kbs downstream of exon 23 and 130 kbs upstream of exon 27, resulting in deletion of exons 24 to 26 (Fig. S1a,b). We next investigated whether the trap vector integration affected the expression of Diap2 protein in B210 ES cells. While Diap2 protein was detected in wild-type ES cells, Diap2 protein completely disappeared in B210 cells (Fig. S1c). This suggests that truncated mRNA of *Diap2* is unstable, causing complete depletion of Diap2 protein. Taken into account that *Diap2* (that is, *Dia3*)-deficient mice develop without abnormalities and are fertile^11^, we reckoned that integration and expression of a target gene in the *Diap2* locus on X chromosome might be feasible by using *Cre-loxP* technology; and further, that this approach would allow us to express the target gene in a mosaic pattern due to random inactivation of one of the two X chromosomes in females.

**Figure 1:**
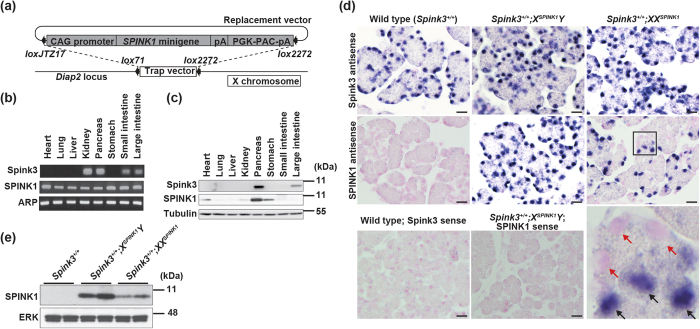
Generation of human *SPINK1* X-chromosome knock-in (“*X-SPINK1*”) mice. (**a**) A replacement vector containing *SPINK1* minigene under the CAG promoter was introduced into *Diap2* locus on the X chromosome by Cre-*lox*P technology. (**b**) RT-PCR analysis of *Spink3* and *SPINK1* mRNAs in various tissues of *X-SPINK1* mice at 8 weeks. Acidic ribosomal phosphoprotein P0 (ARP), a “housekeeping” gene control. (**c**) The levels of Spink3 and SPINK1 proteins in various tissues of *X-SPINK1* mice at 8 weeks (immunoblot). Representative of two independent experiments. (**d**) ISH analysis of *SPINK1* mRNA expression in pancreas of mice of the indicated genotype at P0.5. Pancreatic tissue sections were hybridized with *Spink3* (upper panels) or *SPINK1* (middle panels) antisense riboprobes (blue stain). Nuclei stained with Nuclear Fast Red have a pink appearance. The bottom right panel is an enlarged image of the boxed area in the panel above. Black and red arrows indicate acinar cells with and without *SPINK1* expression, respectively. The bottom left and center panels show ISH background control using *SPINK1* and *Spink3* sense riboprobes. Scale bars, 20 μm. Representative of two independent experiments. (**e**) Pancreas homogenates from mice of the indicated genotype at 8 weeks were analyzed by immunoblot analysis. In this and other figures, ERK or tubulin serve as loading control; each lane represents an individual animal; and the numbers to the right are protein molecular mass markers in kDa.

To this end, we generated a replacement vector containing CAG promoter-human *SPINK1* minigene^12^ flanked by two mutated *lox*P sites, and co-transfected this vector along with a *Cre* recombinase expression vector^13^ into B210 ES cells (Fig. 1a). We obtained 11 ES cell lines harboring *SPINK1* minigene on X chromosome with high frequency, and used these ES cells to generate mice termed “*X-SPINK1*”. Male and female *X-SPINK1* mice were healthy, fertile, and did not show any abnormalities. These knock-in mice are henceforth referred to as, respectively, *X*^*SPINK1*^*Y* (male)*, X*^*SPINK1*^*X*^*SPINK1*^ (female), and *XX*^*SPINK1*^ (female) mice. We first verified the expression of *SPINK1* mRNA and protein in *X*^*SPINK1*^*Y* mice by RT-PCR and immunoblot (IB) analysis. Because *SPINK1* expression in these mice is driven by the ubiquitous CAG promoter, *SPINK1* mRNA was ubiquitously expressed in various tissues we examined (Fig. 1b). Interestingly, SPINK1 protein expression was restricted to pancreas and, to a much lesser extent, stomach and heart (Fig. 1c). The endogenous *Spink3* mRNA expression is restricted to kidney, pancreas, small and large intestines (Fig. 1b), whereas Spink3 protein is predominantly expressed in the pancreas (and to a much lesser extent, large intestine; Fig. 1c).

We next analyzed the expression of *SPINK1* mRNA in pancreas of *X-SPINK1* mice at 0.5 days after birth (P0.5) by *in situ* hybridization (ISH; Fig. 1d). As expected, all pancreatic acinar cells expressed *SPINK1* mRNA in *X*^*SPINK1*^*Y* mice (the wild type *Spink3*^+*/*+^ mice served as negative control). Notably, in *XX*^*SPINK1*^ mice approximately half of acinar cells expressed *SPINK1* mRNA (Fig. 1d), consistent with the mosaic pattern of *SPINK1* mRNA expression. The endogenous *Spink3* was expressed in all pancreatic acinar cells in *X*^*SPINK1*^*Y* and *XX*^*SPINK1*^ mice, same as in *Spink3*^+*/*+^ mice (Fig. 1d). The sense *Spink3* or *SPINK1* riboprobes were used as a background ISH control and did not show significant staining in any of the mouse strains examined. Consistent with mRNA expression, the level of SPINK1 protein in pancreas of *XX*^*SPINK1*^ mice was one-third to one-half of those in *X*^*SPINK1*^*Y* mice (Fig. 1e).

The data in Fig. 1 indicate that integration of *SPINK1* gene on one of the two X chromosomes results in a mosaic pattern of *SPINK1* expression through X-chromosome inactivation.

### Crossing *Spink3* deficient mice with *X-SPINK1* rescues the resultant *Spink3* ^−*/*−^ *;XX* ^*SPINK1*^ mice from perinatal lethality

**Figure 2:**
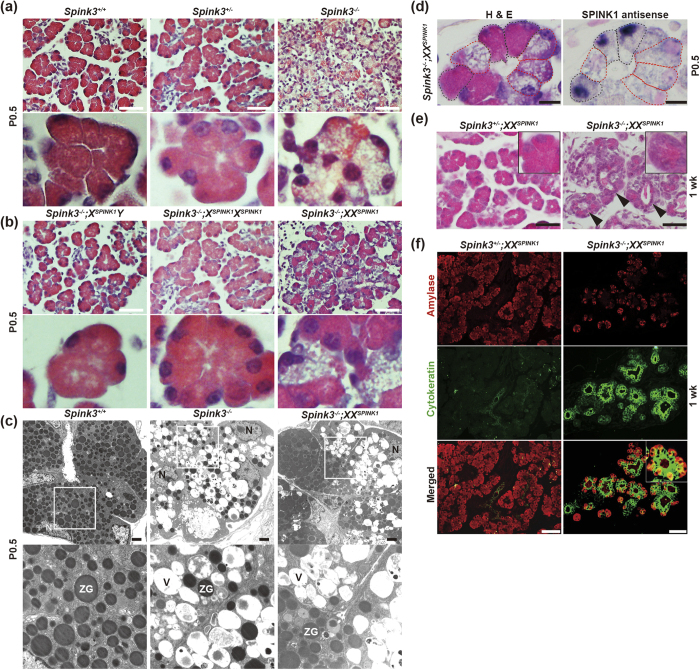
Crossing *Spink3* deficient mice with *X-SPINK1* rescues the resultant *Spink3*^−/−^*;XX*^*SPINK1*^ mice from perinatal lethality. (**a,b**) Hematoxylin and eosin (H&E)-stained pancreatic tissue sections from mice of the indicated genotype at P0.5. The lower rows in (**a**,**b**) show enlarged images of the upper panels. Scale bars, 50 μm. (**c**) Electron micrographs of pancreata of mice of the indicated genotype at P0.5. The lower row shows enlarged images of the areas designated by white boxes in the upper panels. ZG, zymogen granule; V, vacuole; N, nucleus. Scale bars, 2 μm. (**d**) Adjacent pancreatic tissue sections from *Spink3*^−/−^*;XX*^*SPINK1*^ at P0.5 were analyzed by H&E staining and ISH. Acinar cells expressing *SPINK1* are delineated by black dashed lines, and those with no *SPINK1* mRNA expression, by red dashed lines. Scale bars, 10 μm. (**e**) H&E staining of pancreatic tissue sections from mice of the indicated genotype at 1 week. Arrowheads indicate tubular structures. Insets show 2.5x-enlarged areas. Scale bars, 50 μm. (**f**) Cells positive for both amylase and pan-cytokeratin in pancreas of *Spink3*^−/−^;*XX*^*SPINK1*^ mice at 1 week. Pancreatic tissue sections from mice of the indicated genotype were immunostained using anti-amylase (red) or anti-pan-cytokeratin (green) antibodies. Scale bars, 50 μm.

*Spink3*^−/−^ mice die perinatally^3^, making it impossible to investigate long-term effects of *Spink3* deficiency. To circumvent this problem, we crossed *Spink3*^+*/*−^ mice with *X-SPINK1* mice. As we previously reported^3^, pancreatic acinar cells of *Spink3*^−*/*−^ mice exhibited prominent vacuolization at P0.5, which is not seen in pancreas of *Spink3*^+*/*−^ mice (Fig. 2a). The acinar cell vacuolization was prevented in *Spink3*^−*/*−^*;X*^*SPINK1*^*Y* and *Spink3*^−*/*−^*;X*^*SPINK1*^*X*^*SPINK1*^ mice (Fig. 2b), suggesting that ectopic expression of *SPINK1* gene completely compensated for a defect caused by *Spink3* deficiency. Notably, in *Spink3*^−*/*−^*;XX*^*SPINK1*^ mice at P0.5 approximately half of all acinar cells exhibited vacuolization, whereas the other half appeared normal (Fig. 2b). We further used transmission electron microscopy to examine acinar cell morphology in *Spink3*^−*/*−^ and *Spink3*^−/−^*;XX*^*SPINK1*^ mice at P0.5 in greater detail. The cytoplasmic vacuolization was prominent in all acinar cells in *Spink3*^−/−^ pancreas, whereas in the pancreas of Spink3^−/−^*;XX*^*SPINK1*^ mice approximately one-half of acinar cells exhibited vacuolization and the rest appeared normal (Fig. 2c). As we previously reported^3^,^14^,^15^, the fact that large numbers of these vacuoles contain cytoplasmic constituents, in particular organelle remnants, indicates their autophagic nature. Further, many vacuoles contain poorly/incompletely degraded cargo, suggesting a defect in later stages of the autophagy process.

Of note, the histologically normal (i.e., without vacuoles) acinar cells in P0.5 *Spink3*^−/−^*;XX*^*SPINK1*^ pancreas expressed *SPINK1* mRNA as shown by ISH (Fig. 2d; delineated by black dashed line); in contrast, acinar cells that exhibited massive vacuolization did not express *SPINK1* mRNA (Fig. 2d; delineated by red dashed line). Together with our previous findings^3^, these results indicate that the one-half of acinar cells in *Spink3*^−/−^*;XX*^*SPINK1*^ mice that do not express *SPINK1* (due to X-chromosome inactivation) develop prominent vacuolization whereas the other half, which do express *SPINK1*, are devoid of these vacuoles. Surprisingly, acinar cells filled with numerous vacuoles in pancreas of *Spink3*^−/−^*;XX*^*SPINK1*^ mice at P0.5 disappeared at one week after birth (Fig. 2e), suggesting that such cells might have been eliminated during this period. How exactly these cells die out remains to be determined; with TUNEL, we did not detect apoptotic acinar cells in either *Spink3*^−/−^ or *Spink3*^−/−^;*XX*^*SPINK1*^ mice (Fig. S2).

Of note, the number of ductal-like cells forming so-called “tubular structures” dramatically increased in pancreas of 1-week-old *Spink3*^−/−^*;XX*^*SPINK1*^ mice (Fig. 2e). Moreover, these cells were positive for both amylase and cytokeratins (Fig. 2f), suggesting they derived from *SPINK1*-positive acinar cells through acinar-ductal metaplasia^16^.

### *Spink3* ^−/−^ *;XX* ^*SPINK1*^ mice spontaneously develop pancreatitis characterized by chronic inflammation and fibrosis

**Figure 3:**
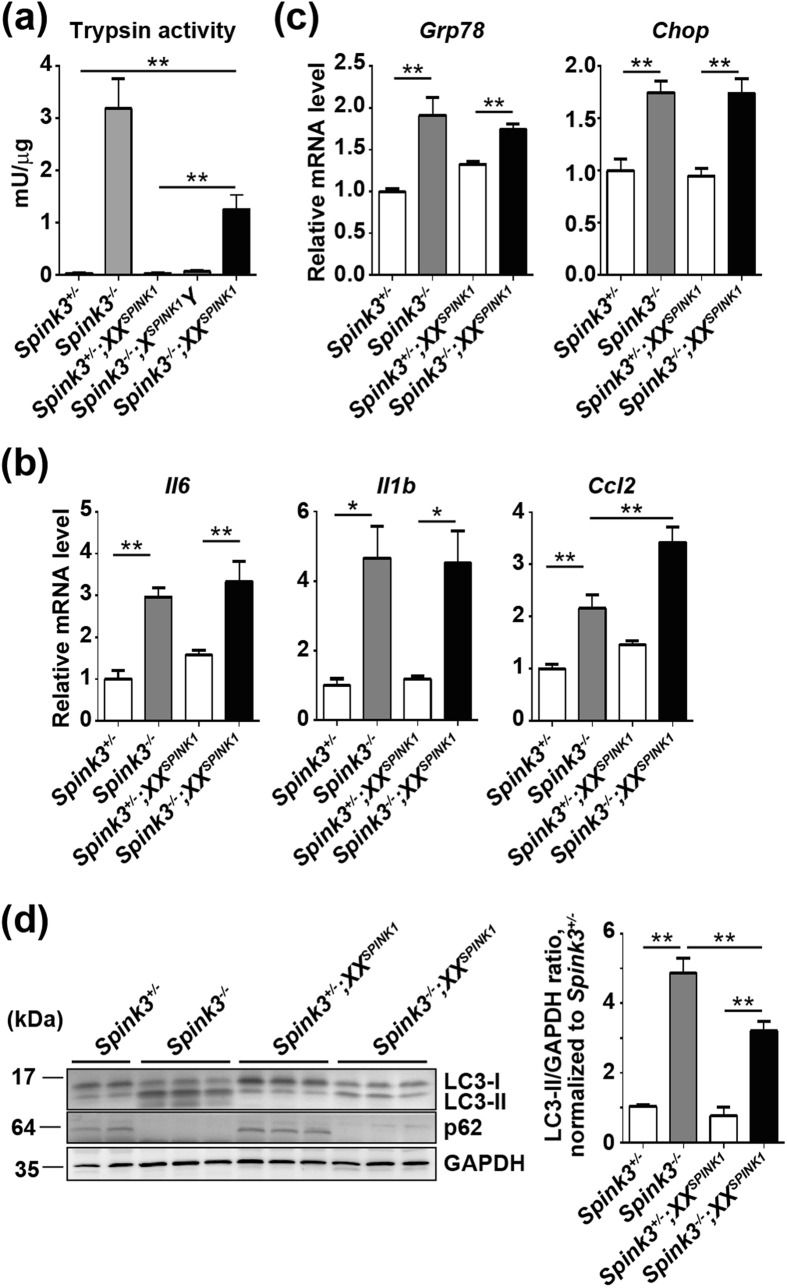
Pancreas damage in *Spink3*^−/−^;*XX*^*SPINK1*^ mice at birth. (**a**) Trypsin activity in pancreas of mice of the indicated genotype at P0.5 was measured by a fluorogenic enzymatic assay (see Methods). Values are means ± SEM (*n* = 4–6 mice). **P < 0.01. (**b,c**) mRNA expression of proinflammatory cytokines and chemokines, and ER stress markers in pancreas of mice of the indicated genotype was analyzed by qPCR. Values are means ± SEM (*n* = 4 mice). *P < 0.05; **P < 0.01. (**d**) Autophagy markers LC3 and p62/SQSTM1 were analyzed by immunoblotting in pancreas of mice of the indicated genotype. The LC3-II/LC3-I band intensity ratio was measured by densitometry and normalized to that in *Spink3*^+/−^ pancreas. Values are means ± SEM (n = 4–6 mice). **P < 0.01.

We investigated the development of pancreatic damage, and the underlying mechanisms, in Spink3^−/−^ and *Spink3*^−/−^*;XX*^*SPINK1*^ mice. The inappropriate increase in pancreatic trypsin activity is a hallmark of both human and experimental pancreatitis^17^,^18^. We find that pancreatic trypsin activity was dramatically upregulated in *Spink3*^−/−^ mice at P0.5 (compared to wild type or *Spink3*^+*/*−^ mice) and that this increase was ~3 times less in *Spink3*^−/−^*;XX*^*SPINK1*^ mice (Fig. 3a). Moreover, there was essentially no increase in pancreatic trypsin activity in *Spink3*^−/−^*;X*^*SPINK1*^*Y* mice (Fig. 3a), indicating that human SPINK1 can compensate for the loss of trypsin inhibition in *Spink3*^−/−^ mice.

Proinflammatory cytokines, such as *Il6* and *Il1b*, and the chemokine *Ccl2/Mcp-1* (monocyte chemotactic protein-1) were upregulated in pancreas of *Spink3*^−/−^ and *Spink3*^−/−^;*XX*^*SPINK1*^ mice at P0.5 (Fig. 3b). In addition, the expression of genes associated with ER stress, such as *Bip/Grp78* and *Chop*, was upregulated in pancreas of *Spink3*^−/−^ and *Spink3*^−/−^;*XX*^*SPINK1*^ mice compared to *Spink3*^+/−^ or *Spink3*^+/−^*;XX*^*SPINK1*^ mice (Fig. 3c). In these (and other) measurements we used *Spink3*^+/−^*;XX*^*SPINK1*^and *Spink3*^+/−^ mice as controls for, respectively, *Spink3*^−/−^;*XX*^*SPINK1*^ and *Spink3*^−/−^ to analyze the effects of mosaic expression of *SPINK1* in rescuing *Spink3*^−/−^ mice from perinatal lethality and the development of pancreatic injury in *Spink3*^−/−^;*XX*^*SPINK1*^ mice; and because *Spink3*^+/−^ mice have normal phenotype. Other mouse strains which can also serve as controls, such as *Spink3*^−/−^*;X*^*SPINK1*^*Y* or *Spink3*^−/−^; *X*^*SPINK1*^*X*^*SPINK1*^, express *SPINK1* in all cells; as noted, these mice do not show any histological or other abnormalities (Figs 2b and 3a; and data below).

We next measured the changes in autophagy markers in pancreas of *Spink3*^−/−^*;XX*^*SPINK1*^ mice. The conversion of microtubule-associated protein 1 light chain 3 (LC3) from the cytosolic LC3-I to the membrane LC3-II form that uniquely localizes to autophagic vacuoles^7^ was increased in pancreas of *Spink3*^−/−^ and (to a lesser extent) *Spink3*^−/−^*;XX*^*SPINK1*^ mice at P0.5, compared to that in *Spink3*^+/−^ and *Spink3*^+/−^*;XX*^*SPINK1*^ mice (Fig. 3d). These data are consistent with acinar cell vacuolization we find in *Spink3*^−/−^ and *Spink3*^−/−^*;XX*^*SPINK1*^ mice (Fig. 2b–d). At the same time, p62/SQSTM1, a protein which both mediates autophagy and is degraded by autophagy^19^, disappeared in pancreas of *Spink3*^−/−^ and *Spink3*^−/−^*;XX*^*SPINK1*^ mice (Fig. 3d). Of note, a recent study^20^ has shown that p62 accumulates in pancreas of mice with pancreas-specific knockout of the kinase IKKα (*Ikka*^*Δpan*^ mice), mediating the development of spontaneous pancreatitis in this genetic model. Our data in Fig. 3d indicate that, in contrast, p62 does not mediate the development of pancreatitis in *Spink3*^−/−^*;XX*^*SPINK1*^ mice.

**Figure 4:**
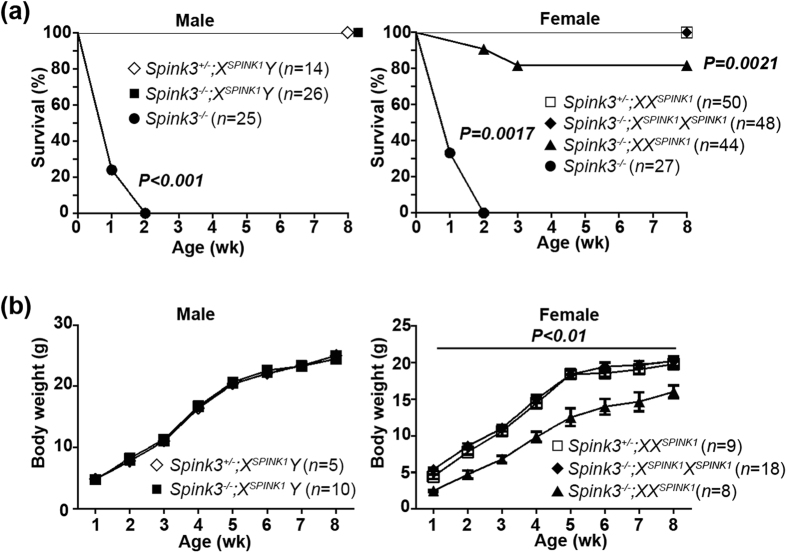
*Spink3*^−/−^;*XX*^*SPINK1*^ mice exhibit mild growth retardation. (**a**) Survival curves of male and female mice of the indicated genotype. *P* value, as compared to the wild type (*Spink3*^+*/*+^), was calculated by the log rank test. (**b**) Body weight gain of mice of the indicated genotype. Values are means ± SEM. *P < 0.05.

**Figure 5:**
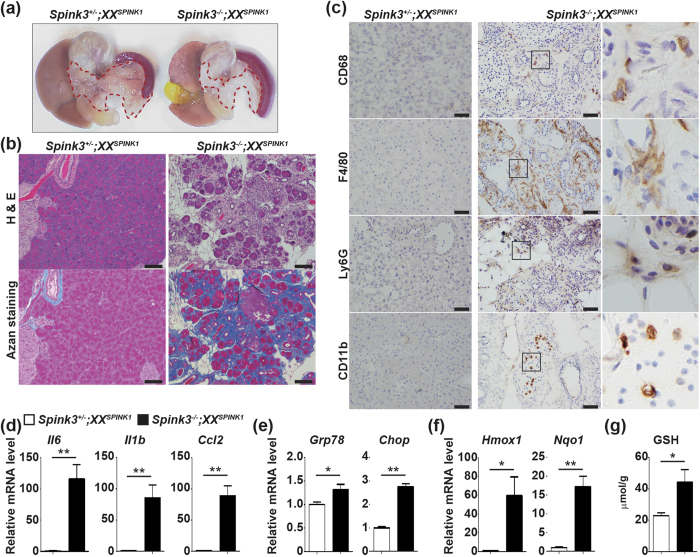
*Spink3*^−/−^;*XX*^*SPINK1*^ mice spontaneously develop chronic pancreatitis. (**a**) Macroscopic analysis of *Spink3*^+/−^*;XX*^*SPINK1*^ and *Spink3*^−/−^;*XX*^*SPINK1*^ mice at 8 weeks. Dashed lines delineate the pancreas. (**b**) Pancreatic tissue sections from mice of the indicated genotype at 8 weeks were stained with H&E (upper panels) or Azan [for collagen (dark blue); lower panels]. Scale bars, 100 μm. (**c**) Pancreatic cryosections from mice of the indicated genotype were stained for immune cell markers CD68, F4/80, Ly-6G, or CD11b (brown). Right-column panels show enlarged images of the boxed areas in the corresponding middle-column panels. Scale bars, 50 μm. (**d–f**) qPCR analysis of gene expression for (**d**) proinflammatory cytokines and chemokines, (**e**) ER and (**f**) oxidative stress markers. (**g**) Reduced glutathione (GSH) levels in pancreas of mice of the indicated genotype at 8 weeks. Values are means ± SEM (*n* = 4–6 mice). *P < 0.05; **P < 0.01.

Although *Spink3*^−/−^ mice die within 2 weeks after birth^3^, all *Spink3*^−/−^*;X*^*SPINK1*^*Y* (male) and *Spink3*^−/−^*;X*^*SPINK1*^*X*^*SPINK1*^ (female) mice grew normally and were healthy at 8 weeks (Fig. 4a,b). Approximately 80% of *Spink3*^−/−^*;XX*^*SPINK1*^ (female) mice survived and exhibited mildly, but significantly, retarded body weight gain compared to *Spink3*^*+/−*^ and *Spink3*^−/−^*;X*^*SPINK1*^*X*^*SPINK1*^ mice (Fig. 4a,b). Macroscopically, the pancreas of *Spink3*^−/−^*;XX*^*SPINK1*^ mice at 8 weeks was atrophic compared to that of *Spink3*^+/−^*;XX*^*SPINK1*^ mice (Fig. 5a). *Spink3*^−/−^*;XX*^*SPINK1*^ mice gradually developed chronic pancreatitis, manifested by loss of acinar cells, intralobular fibrosis, and dilatation of interlobular ducts with protein plugs (Figs 5b and S3, and Supplementary Table 1). In contrast to *Spink3*^−/−^*;XX*^*SPINK1*^ mice, there were no histopathological alterations in pancreas of *Spink3*^−/−^*;X*^*SPINK1*^*Y* and *Spink3*^−/−^;*X*^*SPINK1*^*X* mice even at later age (13 weeks; Fig. S4).

Large number of inflammatory cells including macrophages, neutrophils, and monocytes infiltrated the pancreas, consistent with dramatic upregulation of proinflammatory cytokines and chemokines (Fig. 5c,d). Moreover, the expression of genes induced by ER stress (*Bip/Grp78* and *Chop*), and those induced by oxidative stress (such as *Hmox1* and *Nqo1*) increased in pancreas of *Spink3*^−/−^*;XX*^*SPINK1*^ mice compared to *Spink3*^+/−^*;XX*^*SPINK1*^mice (Fig. 5e,f). Of note, the upregulation of all these genes was much greater at 8 weeks (Fig. 5d–f) than at P0.5 (Fig. 3b,c), illustrating progressive development of pancreatitis in *Spink3*^−/−^*;XX*^*SPINK1*^ mice. The reduced form of glutathione (GSH) protects cellular components from reactive oxygen species such as free radicals and peroxides. The pancreatic GSH level increased in *Spink3*^−/−^*;XX*^*SPINK1*^ mice (Fig. 5g), indicating a protective response.

As stated above (Fig. 4b), body weight of *Spink3*^−/−^*;XX*^*SPINK1*^ mice was somewhat lower than that of *Spink3*^*+/−*^*;XX*^*SPINK1*^and *Spink3*^−/−^*;X*^*SPINK1*^*X*^*SPINK1*^ mice, suggesting a mild exocrine pancreas dysfunction. We did not observe dramatic changes in blood biochemical parameters of *Spink3*^−/−^*;XX*^*SPINK1*^ mice at 8 weeks (Fig. S5), except for 50% decrease in blood triglycerides. Glucose level in blood was not altered; a modest decrease in serum amylase (Fig. S5) may reflect loss of acinar tissue.

### Precancerous changes in the pancreas of *Spink3* ^−/−^;*XX* ^*SPINK1*^mice

**Figure 6:**
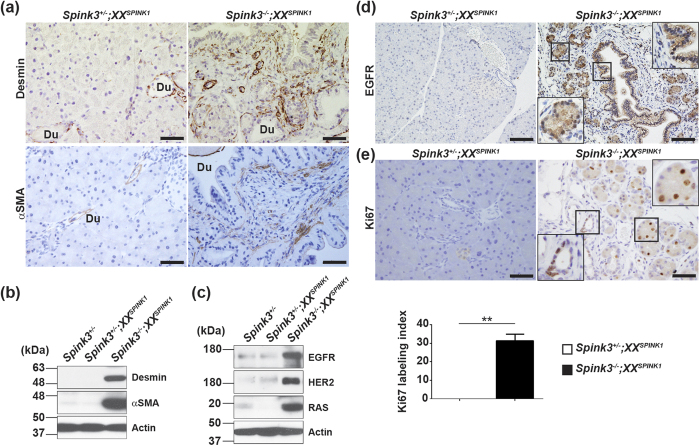
Pre-cancerous changes in pancreas of *Spink3*^−/−^;*XX*^*SPINK1*^ mice. (**a**) Pancreatic tissue sections from mice of the indicated genotype at 8 weeks were immunostained for desmin or αSMA (brown). Scale bars, 50 μm. Du, duct. (**b,c**) Up-regulation of (**b**) stellate cell markers and (**c**) proto-oncogene products in pancreas of *Spink3*^−/−^;*XX*^*SPINK1*^ mice at 8 weeks (immunoblot). (**d**) Pancreatic tissue sections from mice of the indicated genotype at 8 weeks were immunostained for EGFR (brown). Scale bars, 100 μm. (**e**) Ki67 staining of pancreatic tissue sections and Ki67 labeling index at 8 weeks. Scale bars, 50 μm. Values are means ± SEM (*n* = 5 mice). **P < 0.01.

Chronic pancreatitis is associated with a stromal reaction including proliferation and activation of pancreatic stellate cells (PSCs), a type of mesenchymal cells activation of which mediates the development of pancreatic fibrosis^21^. PSCs are desmin-positive; the number of desmin-positive cells greatly increased in pancreas of *Spink3*^−/−^*;XX*^*SPINK1*^ mice compared to *Spink3*^+/−^;*XX*^*SPINK1*^ mice (Fig. 6a), and they accumulated around ducts. We further found increased staining for α-smooth muscle actin (αSMA), a marker of activated PSCs (Fig. 6a). IB analysis (Fig. 6b) confirmed the increases in desmin and αSMA. Moreover, the levels of products of proto-oncogenes implicated in the development of pancreatic cancer, such as EGFR, HER2, and RAS, dramatically increased in pancreas of *Spink3*^−/−^*;XX*^*SPINK1*^ mice at 8 weeks (Fig. 6c). The expression of EGFR was not only detected in acinar-like cells but also in the epithelial cells of hyperplastic ducts (Fig. 6d). Staining for Ki67, a proliferation marker, also dramatically increased in 8-week-old *Spink3*^−/−^*;XX*^*SPINK1*^ mice (Fig. 6e). Together, these data indicate that chronic pancreatitis that develops in *Spink3*^−/−^*;XX*^*SPINK1*^ mice induces pre-cancerous changes, suggestive of PanIN lesions’ formation. However, we did not detect the development of pancreatic adenocarcinoma even in 12-month-old *Spink3*^−/−^*;XX*^*SPINK1*^ mice (data not shown).

## Discussion

In the present study, we have developed a novel method to express a target gene in a mosaic pattern through its’ specific integration onto the X-chromosome by using Cre-*lox*P technology. Consistent with our hypothesis, female *X-SPINK1* mice, in which one of the two copies of X-chromosome is inactivated, express human *SPINK1* mRNA in a mosaic pattern. Crossing *Spink3* deficient mice with *X-SPINK1* rescues the resultant *Spink3*^−/−^*;XX*^*SPINK1*^ mice from perinatal lethality, but they develop chronic pancreatitis. Our results indicate that the one- half of acinar cells in pancreas of *Spink3*^−/−^*;XX*^*SPINK1*^ mice that do not express *SPINK1* (due to X chromosome inactivation) display prominent vacuolization, whereas the other half harboring *SPINK1* on the active X chromosome are histologically normal. In general, the method we have developed should be useful to rescue mice lacking an essential survival gene, and to monitor long-term effects of insufficient gene function.

A number of recent studies have indicated the involvement of lysosomal and autophagic dysfunction in the pathogenesis of pancreatitis, and in particular, the development of chronic inflammation^15^,^20^,^22^,^23^,^24^,^25^,^26^. For example, a recent study^20^ has shown that pancreas specific deletion of *Ikka* causes defective autophagy completion, resulting in accumulation of p62 which mediates the ER and oxidative stresses, leading to chronic inflammation and other pancreatitis responses in the *Ikka*^*Δpan*^ genetic model. In contrast, we here find that p62 protein is dramatically down-regulated in pancreas of *Spink3*^−/−^ and *Spink3*^−/−^*;XX*^*SPINK1*^ mice (at P0.5) and, therefore, is unlikely to mediate the development of chronic pancreatitis in the “*X-SPINK1*” genetic model. Interestingly, recent studies (e.g., ref. 26 and our unpublished data) show that p62 level in pancreas can be regulated not only through its autophagic degradation but also through changes in mRNA expression. Taken together, the accumulation of abnormally large autophagic vacuoles (many of which contain poorly degraded cargo), decrease in p62, and increased LC3-II level suggest that autophagy in pancreas of *Spink3*^−/−^*;XX*^*SPINK1*^ mice might be upregulated but also impaired. Of note, these two effects on autophagy are not mutually exclusive; in particular, both occur in acute cerulein pancreatitis^15^,^24^,^27^. The characterization of pancreatic autophagy in *Spink3*^−/−^*;XX*^*SPINK1*^ mice requires detailed investigation.

Accumulating studies have shown that acute acinar cell injury caused by aberrant activation of trypsin is not sufficient to develop chronic pancreatitis in mice^28^. Also, we did not find evidence for apoptotic death of acinar cells in pancreas of *Spink3*^−/−^*;XX*^*SPINK1*^ mice; it remains to be determined how exactly the cells that do not express *SPINK1* are eliminated in these mice. Our findings presented in another study (manuscript in preparation) indicate contribution of RIPK3-dependent necroptosis^29^ to this process. In addition to loss of acinar tissue, chronic pancreatitis is characterized by persistent inflammation and fibrosis, along with tissue remodeling^5^. Activated PSCs are considered critical for the development of fibrosis in chronic pancreatitis, by producing extracellular matrix proteins as well as proinflammatory cytokines and chemokines^5^. Importantly, we found positive staining for αSMA, a PSCs activation marker, indicating that PSCs mediate fibrosis in *Spink3*^−/−^*;XX*^*SPINK1*^ mice. Thus, *Spink3*^−/−^*;XX*^*SPINK1*^ mice reproduce key responses of human chronic pancreatitis. Further, chronic pancreatitis that develops in *Spink3*^−/−^*;XX*^*SPINK1*^ mice is associated with increases in proliferation and pancreatic levels of proteins implicated in the development of pancreatic cancer, such as EGFR, HER2, and RAS.

In conclusion, we have developed a novel method thereby mice lacking a gene essential for cell survival can be rescued by expressing this gene on the X chromosome. We applied this method, utilizing X-chromosome inactivation, to generate *Spink3*^−/−^*;XX*^*SPINK1*^ mice in which perinatal lethality is rescued and SPINK1 function is partially restored. These mice develop spontaneous pancreatitis, revealing a critical role of SPINK1 in regulating normal autophagy in the exocrine pancreas. Our results, together with recent findings from our groups as well as others^3^,^14^,^15^,^20^,^24^,^26^,^27^, indicate that defects in autophagy function lead to pancreatitis.

## Methods

### Antibodies

The antibodies used in this study were against insulin (Santa Cruz; SC; sc-9168), amylase (SC; sc-12821), Dia3 (SC; sc-10892), glucagon (Dako, A0565), desmin (Dako, M0760), αSMA (Dako, A0851), cytokeratin (Nichirei, 422061), LC3 (Cell Signaling Technology; CST, #2775), p62 (CST, #5114), ERK (CST, #9102), Spink1 (CST, #2744), CD68 (Serotec, MCA1957), CD11b (eBioscience, 11-0112-81), F4/80 (eBioscience, 14-4801-81), Ly-6G (TONBO biosciences, 70-5931), Ki-67 (Novocastra, NCL-Ki67p), EGFR (Proteintech, 18986-1-AP), HER2 (Proteintech, 18299-1-AP), SPINK1 (Abnova, H00006690-M01), Ras (Millipore, #05-516), glyceraldehyde-3-phosphate dehydrogenase (Abcam, ab8245), actin (Sigma, 20-33), and tubulin (Sigma, T5168).

### Animal use

Mice were kept under specific-pathogen-free (SPF) conditions with free access to food and water in a 12 hours light/dark cycle. *Spink3*^−/−^ mice were described previously^3^. C57BL/6 N mice were purchased from the Clea Japan. All animal experiments were performed with the approval of the Kumamoto University Institutional Animal Care and Use Committee (A27-092). All methods were carried out in accordance with the relevant guidelines of the Kumamoto University, including any relevant details.

### Generation of *SPINK1* minigene X-chromosome knock-in mice

*SPINK1* minigene was described elsewhere^12^. For the present study, we have generated a replacement vector^30^ containing *lox*JTZ17-CAG-promoter (pCAGGS)- *SPINK1* minigene-polyA (pA)- phosphoglycerate kinase-1 promoter (PGK)-puromycin resistance gene (PAC)-pA (PGK-PAC-pA)-*lox*2272. The TT2 ES cell line contains a knocked-out *Diap2* allele located on X-chromosome, in which *Diap2* was targeted by the PGK-neomycin resistance gene (neo) fragment flanked by the mutant lox sites, *lox*71 and *lox*2272 (gene trap vector) (Fig. 1a). For co-electroporation with pCAGGS-Cre (a Cre recombinase gene expression vector^13^) and replacement vector plasmids, we used 20 μg of each plasmid and 1 × 10^7^ *Diap2*^+/−^ ES cells. The cells were co-electroporated using a Bio-Rad Gene Pulser, and then cultured for 48 hours in a standard medium supplemented with 2 μg/ml puromycin (Sigma). Selection was maintained for 5 days, and then colonies were picked into 48-well plates and expanded for storage. The puromycin-resistant colonies were analyzed by Southern blotting and by PCR to select ES cell lines showing successful integration of pCAGGS-*SPINK1* minigene-pA sequence. Positive clones were aggregated with ICR morula according to the previously described protocol^30^. Germline transmission was obtained in three mouse lines, resulting in *X-SPINK1* mice, which were back-crossed onto C57BL/6 N mice for at least 5 generations.

### Reverse transcriptase (RT)-PCR analysis

Total RNA was isolated using the RNeasy Mini Kit (Qiagen). cDNA was synthesized using qPCR RT Kit (Toyobo). For the detection of *Ccl2* (Mm00441242), *Il6* (Mm00446190), and *Il1b* (Mm00434228) mRNA, TaqMan Gene Expression Assays on the AB 7500 Real Time PCR System (Applied Biosystems) was used. Other PCR primers are described in the Supplementary Table 2. Densitometric quantification was done using Image J software (http://rsb.info.nih.gov/ij/).

### *In situ* hybridization (ISH)

Pancreases were fixed in 4% paraformaldehyde phosphate buffer solution for 48 hours at room temperature and cut into 4-μm sections. The sections were mounted on glass slides and processed sequentially according to a standard protocol. To prepare the digoxigenin (DIG)-labeled RNA sense (control) and antisense riboprobes (Roche), total RNA was extracted from human or mouse pancreas and then each complementary DNA (cDNA) template was amplified using reverse transcriptase (RT)-PCR. Primers applied in the RT-PCR reactions were as follows: primer hPSTI1 (CGTGGTAAGTGCGGTGCAGT) located in the first exon of *SPINK1* gene; primer hPSTI2 (CGCGGTGACCTGATGGGATT) located in the fourth exon of *SPINK1* gene, and primer mPsti1 (AGTTCTTCTGGCTTTTGCACCC) located in the first exon of *Spink3* gene; primer mPsti25 (TTCAACGAACCCACGTTGCCTT) located in the fourth exon of *Spink3* gene. ISH analysis was performed with a Ventana XT System Discovery (Roche). Nuclear Fast Red staining was performed after ISH.

### Histological and Immunohistochemical analysis

For histological analysis, tissues were fixed overnight in 15% formalin (Wako), embedded in paraffin, sectioned, and stained using standard procedure for H&E, Azan, or Sirius red. Immunohistochemistry was performed by using the antibodies listed above.

### Transmission electron microscopy

Anesthetized mice were fixed by intracardial perfusion with 2% glutaraldehyde and 2% paraformaldehyde in 0.1 M phosphate buffer, pH 7.4. Slices of thus fixed tissues were postfixed with 2% OsO4, dehydrated in ethanol and embedded in Epok 812 (Okenshoji Co.). Ultrathin sections were cut with a ultramicrotome (ultracut N or UC6: Leica), stained with uranyl acetate and lead citrate, and were examined on a Hitachi HT7700 or JEOL JEM-1230 electron microscope.

### Immunoblot analysis

Pancreas or other tissues were disrupted in the Multi-Beads Shocker system (Yasui-Kikai), and homogenized in a RIPA buffer [150 mM M NaCl, 50 mM Tris-HCl (pH 7.2), 1% deoxycholic acid, 1% Triton X-100, 0.1% SDS, 1 mM phenylmethylsulfonyl fluoride, and protease inhibitor cocktail (1:100 dilution; Nacalai tesque)]. The homogenates were subjected to SDS-PAGE and transferred onto Immobilon polyvinylidene difluoride membranes (Millipore). The membranes were blotted using the indicated antibodies, developed with ECL Western Blotting Detection Reagents, and analyzed in a LAS4000 (GE Healthcare Life Sciences). Quantification of the LC3-II to LC3-I ratio was performed with ImageJ software.

### Trypsin activity

To measure trypsin activity, mouse pancreas was disrupted using the Multi-Beads Shocker system (Yasui Kikai), and in buffer containing 5 mM 2-Morpholinoethanesulfonic acid (pH 6.5), 1 mM MgSO_4_, and 250 mM sucrose. Trypsin activity in homogenates was measured fluorimetrically using Boc-Gln-Ala-Arg-MCA (Peptide Institute. Inc.) as a substrate, in the Infinite 200 PRO (Tecan), according to the method of Kawabata *et al.*^31^. Trypsin activity in each sample was determined using a standard curve for purified trypsin (T1426, Sigma).

### Terminal deoxynucleotidyl transferase–mediated deoxyuridine triphosphate nick-end labeling (TUNEL) assay

This was performed by using *in situ* apoptosis detection kit (Wako).

### GSH content measurement

Pancreas were homogenized in 5% methacholic acid buffer. After homogenization, the lysates were centrifuged at 2800 g and the supernatants were collected and used for GSH assays. Determination of GSH was performed with the GSH assay kit (Oxis International) according to the manufacturer’s instructions. To normalize GSH contents per mg protein of liver extracts, we solubilized the pellets in a RIPA buffer and measured protein content with the Bradford assay (Pierce).

### Statistical analysis

Data in graphs are expressed as means ± standard error of mean (SEM) from 4 or more experiments per group, and each experiment was performed at least twice. Statistical analysis was performed by using unpaired Student’s t test or one-way analysis of variance (ANOVA) test, as appropriate, with GraphPad Prism 6 (GraphPad Software, Inc.). *P* < 0.05 was considered to be statistically significant.

## Additional Information

**How to cite this article**: Sakata, K. *et al.* Novel method to rescue a lethal phenotype through integration of target gene onto the X-chromosome. *Sci. Rep.* **6**, 37200; doi: 10.1038/srep37200 (2016).

**Publisher’s note:** Springer Nature remains neutral with regard to jurisdictional claims in published maps and institutional affiliations.

## Supplementary Material

Supplementary Information
