# Supplementary material for: Novel method to rescue a lethal phenotype through integration of target gene onto the X-chromosome
Source: Sci Rep. 2016 Nov 15;6:37200. doi: 10.1038/srep37200 (PMC5109027; doi:10.1038/srep37200)
Supplement: Supplementary Information [file srep37200-s1.doc]

**Novel method to rescue a lethal phenotype through integration of target gene onto the X-chromosome**

Kazuya Sakata^1,2^, Kimi Araki^1^, Hiroyasu Nakano^3^, Takashi Nishina^3^, Sachiko Komazawa-Sakon^3^, Shin Murai^3^, Grace E. Lee^4^, Daisuke Hashimoto^2^, Chigure Suzuki^5^, Yasuo Uchiyama^5^, Kenji Notohara^6^, Anna S. Gukovskaya^4^, Ilya Gukovsky^4^, Ken-ichi Yamamura^1^, Hideo Baba^2^, and Masaki Ohmuraya ^1*^

^1^Institute of Resource Development and Analysis, Kumamoto University

^2^Department of Gastroenterological Surgery, Kumamoto University

^3^Department of Biochemistry, Toho University School of Medicine, 5-21-16 Omori-Nishi, Ota-ku, Tokyo 143-8540, Japan

^4^Department of Medicine, David Geffen School of Medicine at the University of California Los Angeles, and VA Greater Los Angeles Healthcare System, Los Angeles, CA, USA

^5^Department of Cellular and Molecular Neuropathology, Juntendo University Graduate School of Medicine, 2-1-1 Hongo, Bunkyo-Ku, Tokyo 113-8421, Japan

^6^Department of Pathology, Kurashiki Central Hospital

**Supplementary materials**


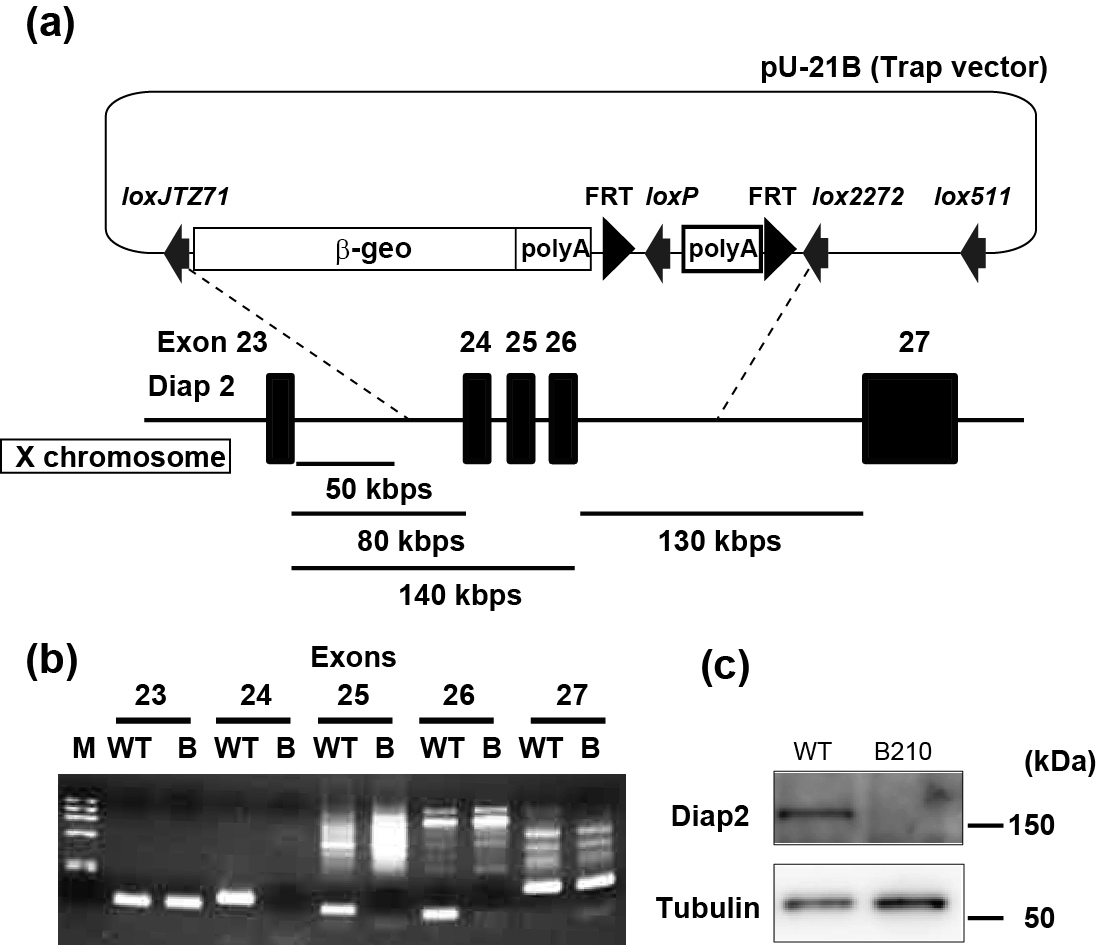


**Supplementary Figure 1** | Generation of *Diap2* knockout ES cell line by gene trap technology. (**a**) Schema of *Disp2* gene knockout by trap vector (pU-21B). (**b**) PCR analysis indicates where trap vector is integrated in *Diap2* gene. (**c**) ES cell lysates were analyzed by immunoblotting. Tubulin served as loading control.


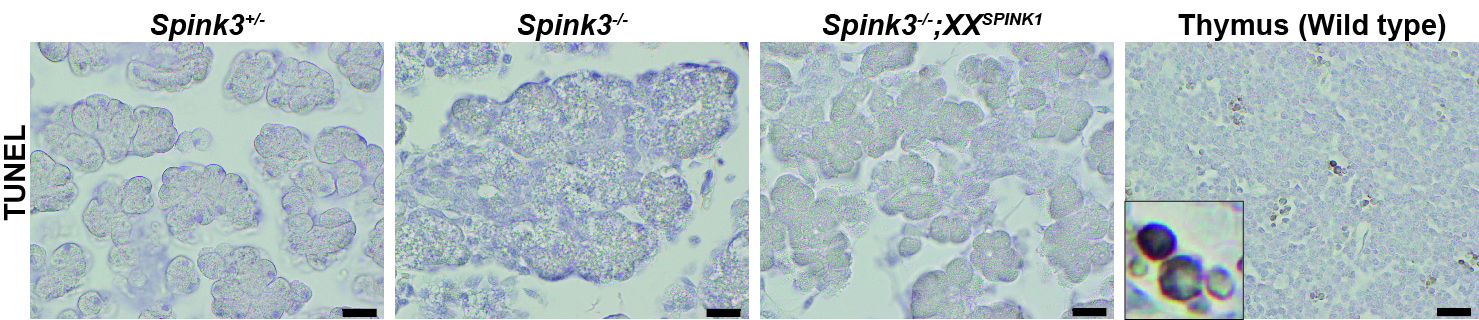


**Supplementary Figure 2** | TUNEL staining of pancreatic tissue sections from mice of the indicated genotype at P0.5. Thymus was used as a positive control (8-week-old wild type mice). Scale bars, 20 μm.


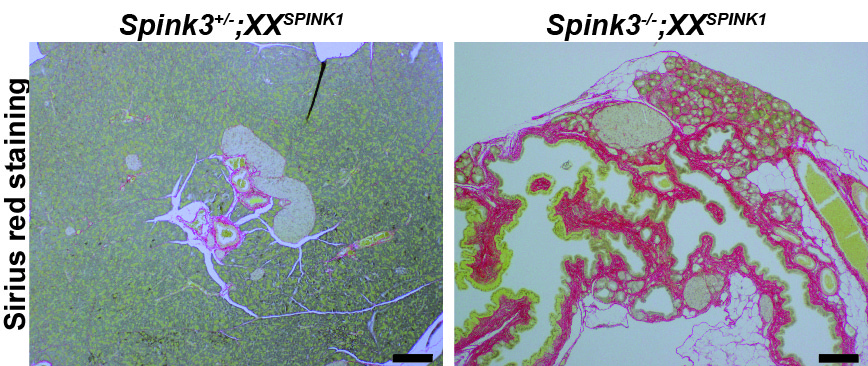


**Supplementary Figure 3** | Prominent fibrosis in pancreas of *Spink3*^-/-^;*XX ^SPINK1^* mice. Pancreatic tissue sections from mice of the indicated genotype at 8 weeks were stained with Sirius red. Scale bars, 200 µm.

**
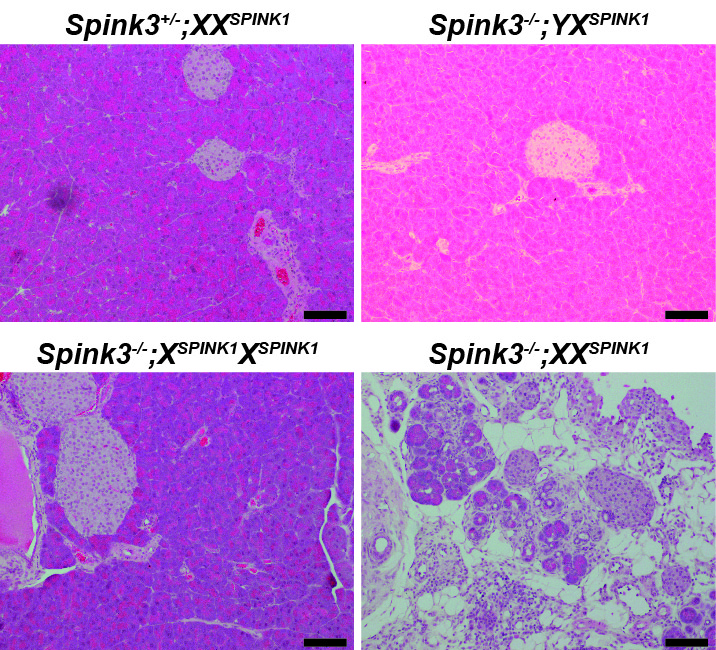
**

**Supplementary Figure 4** | H&E staining of pancreatic tissue sections from mice of the indicated genotype at 13 weeks. In contrast to *Spink3*^-/-^;*XX ^SPINK1^* mice, there are no histopathological alterations in pancreas of mice that express *SPINK1* (or *Spink3*) in all cells. Scale bars, 100 µm.


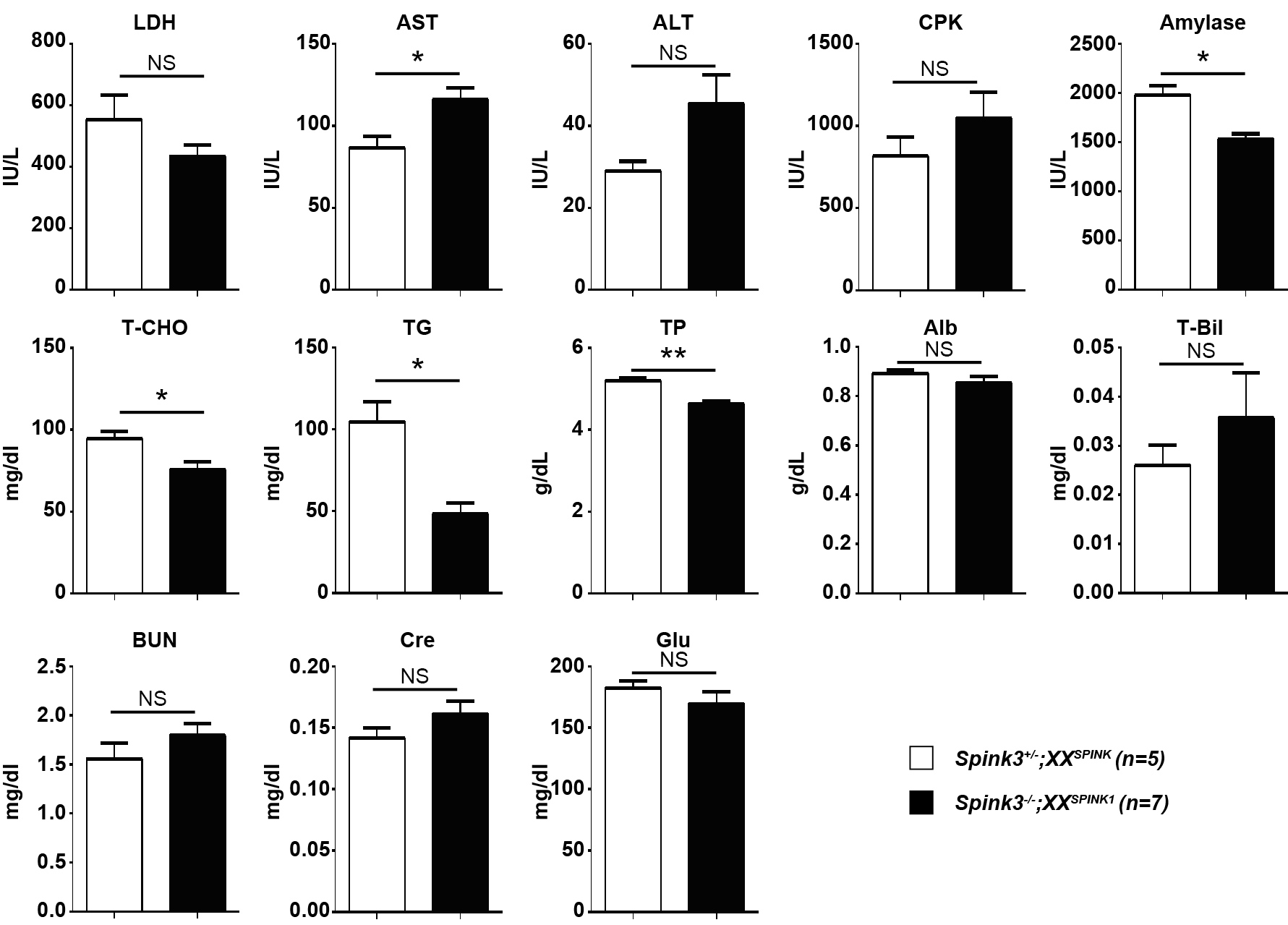


**Supplementary Figure 5** | Blood parameters in *Spink3*^-/-^;*XX ^SPINK1^* mice. Serum markers were determined in mice of the indicated genotype at 8 weeks. LDH, lactate dehydrogenase; AST, aspartate aminotransferase; ALT, alanine aminotransferase; CPK, creatine phosphokinase; T-CHO, total cholesterol; TG, triglycerides; TP, total protein; Alb, albumin; T-Bil, total bilirubin; Cre, creatinine; Glu, glucose. Values are means ± SEM (*n*=5-7 mice). **P*<0.05, ***P*<0.01.


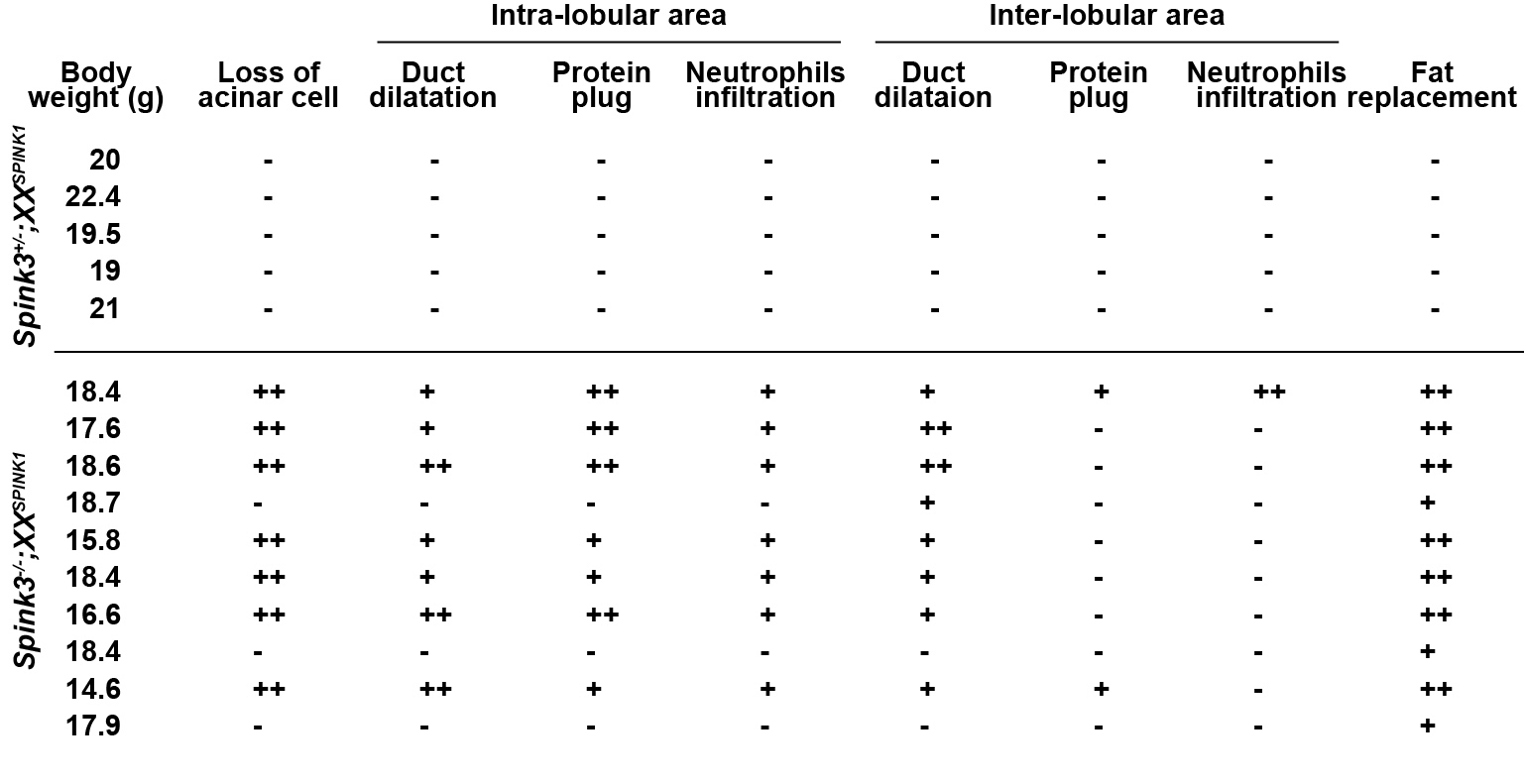


**Supplementary Table 1** | Summary of pathological scores for the pancreata of mice of the indicated genotype at 8 weeks. Each entry represents an individual animal. +, mild; ++, moderate/severe.


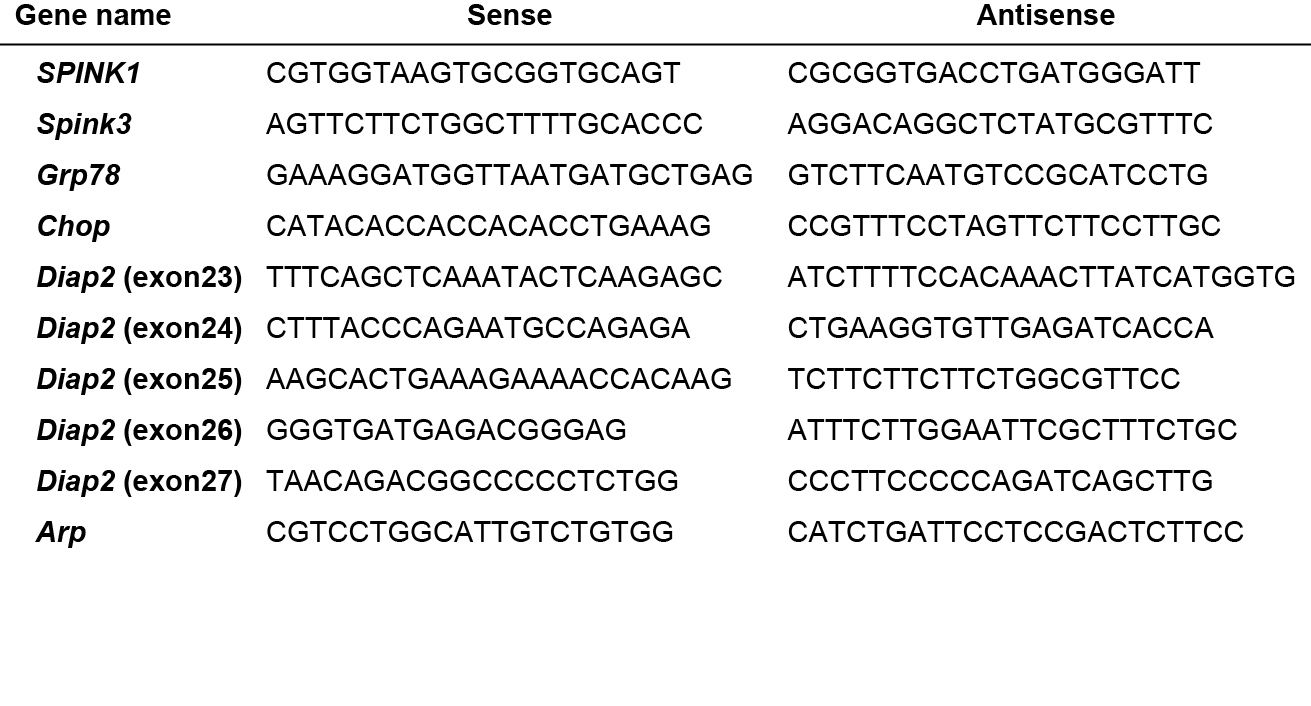


**Supplementary Table 2** | Primers used in the PCR.
